# Inhibition of the Human Hsc70 System by Small Ligands as a Potential Anticancer Approach

**DOI:** 10.3390/cancers13122936

**Published:** 2021-06-11

**Authors:** Leire Dublang, Jarl Underhaug, Marte I. Flydal, Lorea Velasco-Carneros, Jean-Didier Maréchal, Fernando Moro, Maria Dolores Boyano, Aurora Martinez, Arturo Muga

**Affiliations:** 1 Instituto Biofisika (UPV/EHU, CSIC), Universidad del País Vasco, (UPV/EHU), Barrio Sarriena, 48940 Leioa, Spain; leire.dublang@ehu.eus (L.D.); lorea.velasco@ehu.eus (L.V.-C.); fernando.moro@ehu.eus (F.M.); 2 Departamento de Bioquímica y Biología Molecular, Facultad de Ciencia y Tecnología, Universidad del País Vasco, (UPV/EHU), Barrio Sarriena, 48940 Leioa, Spain; 3 Department of Biomedicine, University of Bergen, Jonas Lies vei 91, 5009 Bergen, Norway; jarl.underhaug@uib.no (J.U.); Marte.Flydal@uib.no (M.I.F.); aurora.martinez@uib.no (A.M.); 4 Department of Chemistry, University of Bergen, Allégaten 41, 5007 Bergen, Norway; 5 Departament de Química, Universitat Autònoma de Barcelona (UAB), 08193 Cerdanyola del Vallès, Spain; jeandidier.marechal@uab.cat; 6 Departamento de Biología Celular e Histología, Facultad de Medicina y Enfermería, Universidad del País Vasco, (UPV/EHU), Barrio Sarriena, 48940 Leioa, Spain; lola.boyano@ehu.eus; 7 Instituto de Investigación Biocruces Bizkaia, Hospital Universitario Cruces, 48903 Barakaldo, Spain

**Keywords:** chaperones, drug repurposing, inhibitors, melanoma, pinaverium bromide

## Abstract

**Simple Summary:**

High levels of Heat shock proteins (Hsps) in specific cancers are usually linked to a poor prognosis, tumor progression, invasiveness, and resistance to treatment. Chaperone inhibition could therefore be toxic for cancer cells due to their high dependence on chaperone activity to survive. This study shows the potential to repurpose the small chemical compound pinaverium bromide, currently used to treat functional gastrointestinal disorders, as a possible antitumor drug since it displays a marked toxicity against two melanoma cell lines without affecting the viability of fibroblast and primary melanocytes. This compound interacts with structural regions shared by representatives of the Hsp70 and Hsp110 families, inhibiting the substrate remodeling ability of the Hsp70 system in vitro and in a cellular context.

**Abstract:**

Heat shock protein (Hsp) synthesis is upregulated in a wide range of cancers to provide the appropriate environment for tumor progression. The Hsp110 and Hsp70 families have been associated to cancer cell survival and resistance to chemotherapy. In this study, we explore the strategy of drug repurposing to find new Hsp70 and Hsp110 inhibitors that display toxicity against melanoma cancer cells. We found that the hits discovered using Apg2, a human representative of the Hsp110 family, as the initial target bind also to structural regions present in members of the Hsp70 family, and therefore inhibit the remodeling activity of the Hsp70 system. One of these compounds, the spasmolytic agent pinaverium bromide used for functional gastrointestinal disorders, inhibits the intracellular chaperone activity of the Hsp70 system and elicits its cytotoxic activity specifically in two melanoma cell lines by activating apoptosis. Docking and molecular dynamics simulations indicate that this compound interacts with regions located in the nucleotide-binding domain and the linker of the chaperones, modulating their ATPase activity. Thus, repurposing of pinaverium bromide for cancer treatment appears as a promising novel therapeutic approach.

## 1. Introduction

Molecular chaperones are key components of the proteostasis network that maintain the equilibrium between protein synthesis and degradation. This network regulates protein localization, prevents protein misfolding and aggregation, and keeps the native conformation and function of proteins. It also assists in the clearance of hazardous protein species by directing them to autophagy or proteasomal degradation, and in the reactivation of protein aggregates when their formation cannot be avoided [1]. The collective function of chaperones is essential to maintain cellular homeostasis leading to survival [2,3]. When cells are exposed to physiological and environmental stresses, a significant increase in the synthesis of these evolutionarily conserved and ubiquitously expressed proteins [4,5], also known as heat shock proteins (Hsps), allows the cell to tackle otherwise lethal conditions. In human cells, a combination of proteins of the Hsp40/J-domain protein (JDP), Hsp70 (Hsc70 and Hsp70) and Hsp110 (nucleotide exchange factor; NEF) families constitutes a powerful protein remodeling system that forms part of the cellular protein quality control system [1,2,3,4,5]. The highly versatile Hsp70 system favors different housekeeping activities, including folding of newly synthesized proteins, transport of polypeptides across cellular membranes, assembly/disassembly of protein complexes, and regulation of protein activity. It also performs stress-related cellular repair processes, such as prevention of protein aggregation, refolding of misfolded proteins, and solubilization of aggregated proteins. In addition, it collaborates with cellular degradation machineries to clear damaged proteins. Among the different members of the Hsp70 family, the Hsc70 group is constitutively expressed whereas the Hsp70 group is induced by cellular stress [1,2,3].

The representatives of the Hsp70 and Hsp110 families share their overall structure, which contains two functional domains: the N-terminal nucleotide-binding domain (NDB) responsible for ATP hydrolysis, and the C-terminal substrate-binding domain (SBD). A conserved linker allows interdomain communication in Hsp70 [6]. The Hsp110 chaperone family constitutes an Hsp70 divergent branch and, as compared with the Hsp70 family, it has two main insertions in the SBD: an acidic subdomain and a C-terminal extension [7]. Besides being NEFs for Hsp70s, Hsp110 proteins have the ability to prevent protein aggregation, promote protein folding, and confer thermotolerance to cells and thus function as chaperones themselves [8,9,10]. Hsp70s, in contrast to representatives of the human Hsp110 family, undergo a remarkable conformational change driven by ATP binding and hydrolysis. In the ATP-bound state, the SBD has low affinity for substrates resulting in fast association and dissociation rates. ATP hydrolysis triggers a conformational switch resulting in higher affinity for substrates [11]. This conformational cycle, and, therefore, the chaperone activity of Hsp70s, is tightly regulated through their cooperation with JDPs and NEFs that fine-tune the ATP-dependent binding and release of the protein substrate. The interaction of Hsp70 with a JDP and a client protein accelerates ATP hydrolysis by Hsp70 and allows the efficient trapping and remodeling of the protein substrate [12,13]. Substrate release is modulated by NEFs, as Apg2, which replace ADP by ATP and reset the Hsp70 chaperone cycle allowing binding of a new client [14].

Dysregulation of the expression of cytoprotective Hsps [15] under stress conditions contributes to the development of several diseases including cancer [16]. Cancer cells typically have higher metabolic needs and inappropriately activated signaling pathways compared to normal cells and show a higher demand for chaperone activity to survive. Cancer development involves an increase in oncogene levels by activating mutations in those oncogenes and in tumor suppressor genes [16], whose folding and conformation maintenance requires elevated levels of Hsp [17,18]. High levels of Hsp expression in specific cancers is often related to a poor prognosis and plays an important role in suppression of apoptosis, spontaneous as well as triggered by therapeutic intervention, which aids in tumor progression, invasiveness and resistance to treatment [19,20,21]. Therefore, inhibition of Hsps offers the possibility to target different oncoproteins and signaling pathways [22]. As other Hsp families, Hsp110 and Hsp70 have been related to poor prognosis and malignancy in different types of cancer. Hsp110 overexpression has been linked to the inhibition of apoptosis of transformed cells [23,24,25], cancer progression and chemotherapy resistance [26,27,28]. Similarly, Hsp70 expression has been associated with tumor initiation, progression and survival in a variety of cancer types [29,30]. Moreover, Hsp70 and Hsp110 are integrated in the “epichaperome”, a chaperone network that bridges chaperones to different cellular pathways, and is vital for tumor survival [31].

The aim of this study is to take advantage of drug repurposing to look for potential Hsp110 and Hsp70 inhibitors for cancer treatment. Drug repurposing seeks to generate novel clinical opportunities for known approved drugs [32]. Data from both pre-clinical and clinical trials have clearly demonstrated antitumor efficacy of well-known and well-characterized compounds within a wide range of drug classes beyond anticancer compounds [33]. We chose Apg2, a representative of the human Hsp110 chaperone family, as target for screening based on the finding that overexpression of this protein has been related to different types of cancer [34,35,36]. Despite the importance of this finding, there is only one reported Hsp110 inhibitor, which interacts with a cleft within the ATP binding pocket of the chaperone, as suggested by molecular docking, and inhibits colorectal cancer cell growth [34]. The search for potential Apg2 modulators was carried out by differential scanning fluorimetry (DSF). This technique identifies protein binders as they often induce a shift in the midpoint denaturation temperature (*T*_m_) of the protein of interest upon binding [37]. Ligand binding can induce protein stabilization if the ligand preferentially binds to the native state, or destabilization when it interacts with a state(s) less stable than the native one [38]. In the case of Hsp70s, NMR and computational studies have provided a molecular view of the chaperone functional cycle with multiple conformational states that might bind these ligands with different affinity [39]. If the stability of some of these conformations were lower than that of the native state, ligand binding would result in protein destabilization.

In this work, we screen the Prestwick Chemical Library of small molecule drugs, searching for compounds that interact with Hsc70 and Apg2 chaperones and characterize their effect on the remodeling activity of the Hsp70 system, using DnaJB1 as JDP. Our data indicate that one compound, pinaverium bromide, inhibits the Hsp70 system in vitro and in a cellular context compromising specifically the viability of two melanoma cell lines.

## 2. Results

### 2.1. High-Throughput Screening (HTS) and Validation of Modulators of Apg2

**Figure 1:**
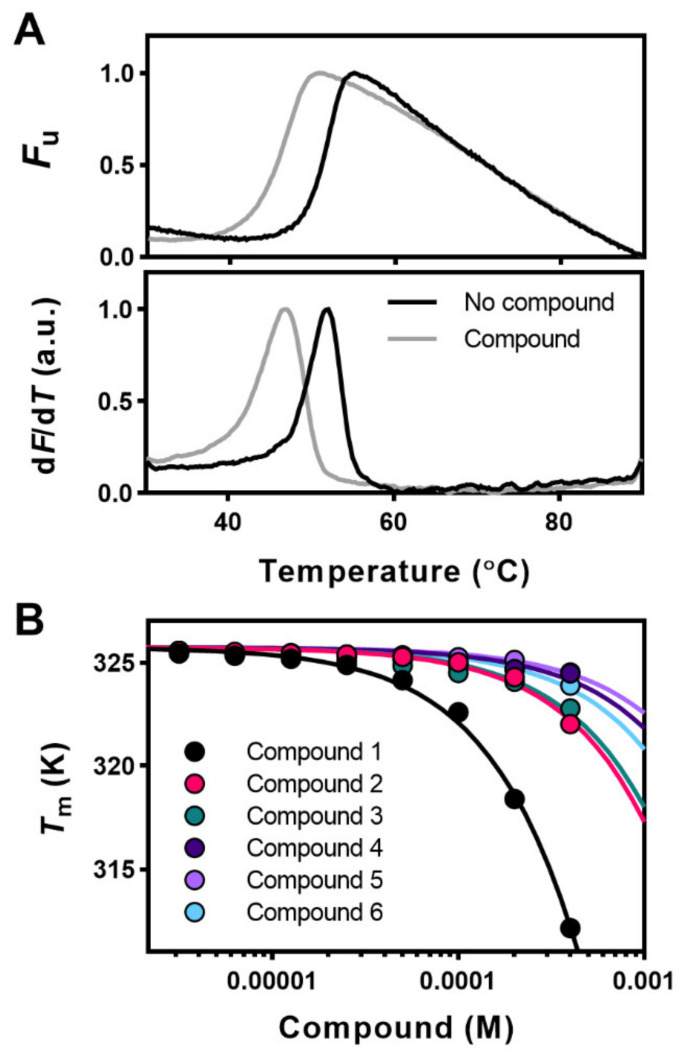
Thermal stability of Apg2 reflects ligand binding. (**A**) Original DSF denaturation curves (upper traces) and first derivative (bottom traces) of a destabilizing (Δ*T_m_* = −3.7 °C) compound. Curves obtained in the absence (reference; black line) and presence of 400 µM of the corresponding compound (grey line). The minimum and maximum fluorescence signals were given the values of 0 and 1, respectively. (**B**) Effect of increasing ligand concentration on the midpoint denaturation temperature (*T_m_*) and shift of the *T_m_* of Apg2. Among the initial hits considered, compounds **1–6** induced a concentration-dependent shift of the midpoint denaturation temperature (Δ*T_m_*) of Apg2. For data fitting, see Materials and Methods. The name, chemical structure, and molecular weight of these compounds are shown in Table 1 and Figure S1.

In this study we screened the Prestwick Chemical Library^®^, composed of 1280 off-patent, 99%-approved drugs (FDA, EMEA and other agencies) selected for their high chemical and pharmacological diversity, to find drugs that could be repurposed [40]. Since all the compounds from the library are dissolved in DMSO, we first addressed whether this solvent would modify the *T*_m_ of Apg2. The thermal stability of the protein was studied by DSF between 30 and 90 °C at the highest DMSO concentrations (4%) used in this study. The maximum from the first derivative of the temperature profile that gave the *T*_m_ values (52.38 ± 0.03 °C without and 51.76 ± 0.25 °C with DMSO, respectively; *n* > 3) showed that the solvent did not significantly affect the thermal stability of the protein. The sample containing this solvent concentration was used as the reference in the screening. Sypro Orange, the fluorescent dye used, is an indicator of protein unfolding as it interacts with hydrophobic patches that the protein exposes during thermal denaturation [37]. Data obtained from the DSF experiments (Figure 1A) showed an initial low fluorescence value that increased within a temperature interval, reaching a maximum fluorescence signal upon protein unfolding. At post-denaturation temperatures, the fluorescence signal decreased probably due to aggregation of the unfolded protein. The 15 compounds that increased or reduced the *T*_m_ value (Δ*T*_m_) ≥ 3-fold × SD of DMSO controls were considered potential hits.

Upon validation of the potential Apg2 binders by the effect of increasing compound concentrations on the *T*_m_ of the protein, only six hits, all destabilizers, showed the expected concentration-dependent change in protein stability (Figure 1B), and they were considered real binders and selected for further studies. The name and chemical structure of these compounds are depicted in Table 1 and Figure S1, respectively. For stabilizing ligands, fitting of the concentration dependent *T*_m_ values to Equation (2) in Cooper and McAuley-Hecht (1993) [41] provides the K_d_ values if the enthalpy of the thermal transition (ΔH_0_) of the protein target is known. However, the complex conformational equilibrium of Hsp110, and of chaperones in general, and the observed destabilizing effect of the selected compounds [38] precluded estimation of the K_d_ values by DSF experiments, and the concentration-dependent *T*_m_-analysis (Figure 1B) was, thus, only used to validate the binders.

**Table 1:** Name, molecular weight, and therapeutic effect of the selected compounds.

Compound	Chemical Name	IUPAC Name	Molecular Weight (Da)	Therapeutic Class
**1**	Chlorhexidine [42]	(1E)-2-[6-[[amino-[(E)-[amino-(4-chloroanilino)methylidene]amino]methylidene]amino]hexyl]-1-[amino-(4-chloroanilino)methylidene]guanidine	505.46	Infectiology
**2**	Pinaverium bromide [43]	4-[(2-bromo-4,5-dimethoxyphenyl)methyl]-4-[2-[2-(6,6-dimethyl-2-bicyclo [3.1.1]heptanyl)ethoxy]ethyl]morpholin-4-ium bromide	591.43	Neuromuscular
**3**	Benzbromarone [44]	(3,5-dibromo-4-hydroxyphenyl)-(2-ethyl-1-benzofuran-3-yl)methanone	424.1	Cardiovascular
**4**	Beta-escin [45]	(beta-D-Xylopyrannosyl)-3(beta-D-glucopyrannosyl)-4(methyl-3acetoxybutyryl)-28tetrahydroxy-16alpha,21alpha,22beta,24oleanone-12	1131.28	Metabolism Oncology
**5**	Mefloquine hydrochloride [46]	(S)-[2,8-bis(trifluoromethyl)quinolin-4-yl]-[(2R)-piperidin-2-yl]methanol hydrochloride	414.78	Infectiology
**6**	Tiratricol, 3,3′,5-triiodothyroacetic acid [47]	2-[4-(4-hydroxy-3-iodophenoxy)-3,5-diiodophenyl]acetic acid	621.94	Endocrinology

### 2.2. Apg2 Binders Also Interact with Hsc70

As mentioned, Apg2 harbors two regions that distinguishes it from Hsc70, namely an acidic subdomain (AS) of 65 residues inserted in the β subdomain of the SBD, and an extension of 141 amino acids at the C-terminus (Figure S2A). Both regions are predicted to adopt mainly an intrinsically disordered structure [48,49] (Figure S2B).

**Figure 2:**
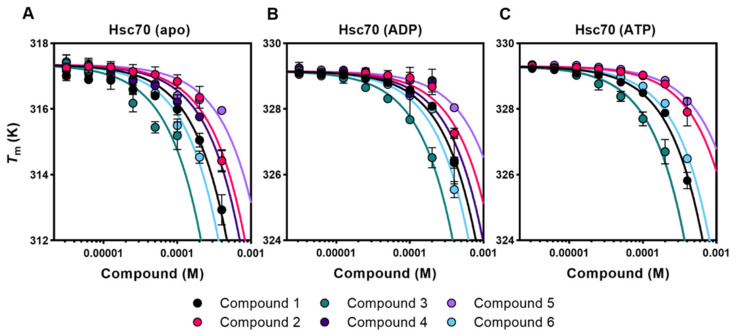
Selected compounds also affect the thermal stability of free and nucleotide-bound Hsc70. (**A**–**C**) Titrations of compounds **1–6** against apo-Hsc70 (**A**), Hsc70-ADP (**B**), and Hsc70-ATP (**C**). For fitting of the data, see Materials and Methods. The name, chemical structure, and molecular weight of these compounds are shown in Table 1 and Figure S1.

To find out whether some of the selected compounds target these intrinsically disordered regions (IDRs), their interaction with two variants of Apg2 that lack one of these characteristic regions, namely Apg2ΔAS and Apg2ΔC, was studied by DSF (Figure S2C). The results showed that although the thermal shifts observed for these deletion mutants were in some cases different, they displayed a similar concentration dependence to the full-length protein, suggesting that these compounds did not target specifically the acidic subdomain and the C-terminal extension of Apg2. It thus appeared that the compounds interacted with protein regions that might well be shared by Hsc70. If this were the case, they could also bind to Hsc70 and, thus, this possibility was also explored with DSF. In contrast to Apg2, Hsc70 undergoes a conformational cycle driven by ATP hydrolysis at the NBD, which involves the allosteric rearrangement of its two domains [6,11]. Therefore, the effect of the selected compounds on the thermal stability of Hsc70 was analyzed in the presence of saturating nucleotide concentrations (ADP- and ATP-states) and in the absence of nucleotide (apo-form). Data showed that the six compounds induced a significant destabilization of Hsc70, especially of its apo-form, in a concentration-dependent manner (Figure 2).

### 2.3. Effect of the Compounds on the Chaperone Activity of Hsc70 and Apg2

**Figure 3:**
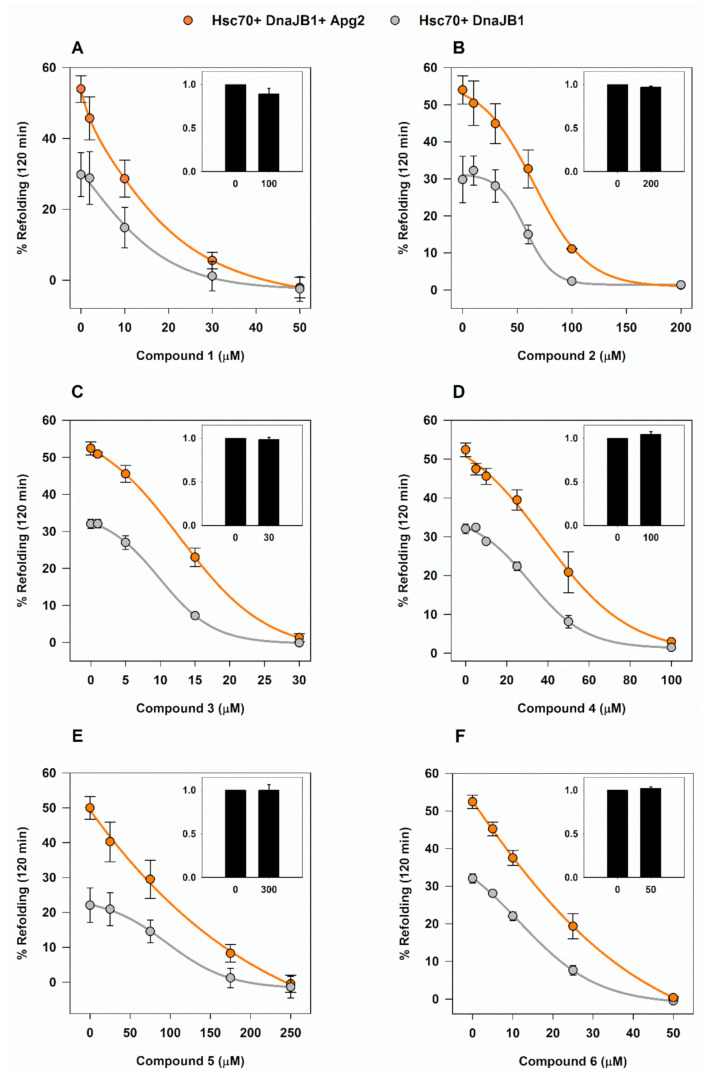
Chaperone-mediated reactivation of G6PDH aggregates in the presence of the selected compounds. (**A**–**F**) The effect of compounds **1–6** on the Hsp70-based disaggregase system was analyzed by following their effectiveness to reactivate G6PDH aggregates (0.4 µM) obtained after denaturing and aggregating the client protein at 50 °C during 30 min. Samples contained 2 µM Hsc70 and 0.5 µM DnaJB1 either without (light grey) or with 0.4 µM Apg2 (orange). Values are shown as the means ± SD of three independent experiments. Insets, effect of the highest compound concentration on the activity of native G6PDH, relative to control samples without compound.

G6PDH was used as a model client protein to follow the chaperone activity of the binary (Hsc70/DnaJB1) and ternary (Hsc70/DnaJB1/Apg2) chaperone mixtures, as they can reactivate aggregates of this substrate protein (Figure S3) and none of the compounds significantly affected the activity of native G6PDH (Figure 3, insets). The addition of up to 2% DMSO (*v*/*v*) did not affect the functionality of the chaperones and, therefore, this was the highest solvent concentration used in these experiments.

Compounds were added to the binary and ternary chaperone mixtures and in all cases, a concentration-dependent inhibition of the refolding was observed after a 2-h assay (Figure 3). As expected, the samples that did not contain Apg2 were also inhibited, supporting the DSF data showing that the compounds also interact with Hsc70. The effect of the compounds differed on the minimum concentration necessary to suppress reactivation of G6PDH aggregates and, hence, on their half maximal inhibitory concentration (IC_50_) values as shown in Table 2.

### 2.4. Toxicity of the Inhibitors in Human Melanoma Cell Lines

Compounds **1–6** inhibited, although with different effectiveness, substrate remodeling by the Hsc70 system in a concentration-dependent manner. Due to the important role of Hsps in the regulation of cancer development [50], we next analyzed whether these compounds affected the growth of cancer cells. With that purpose, their toxicity was assayed using the tumorigenic melanoma cell lines A2058 and MeWo. Among many other types of cancer, melanoma shows a direct relation between Hsp overexpression and disease malignancy [51].

**Figure 4:**
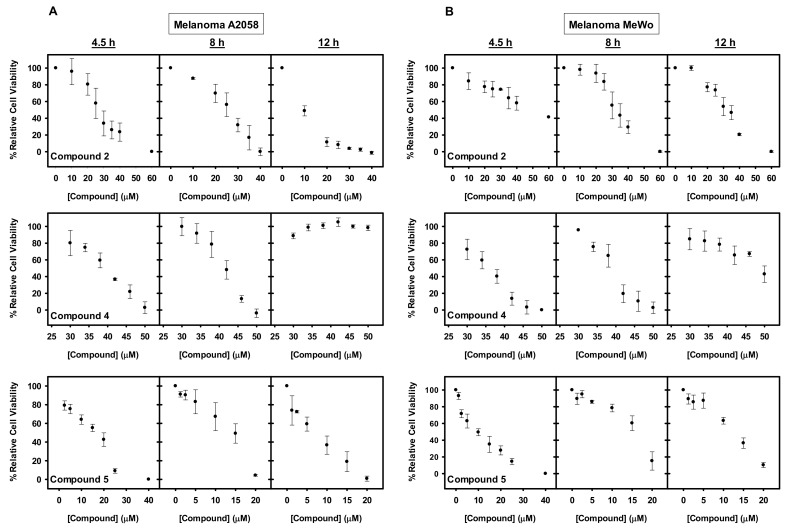
Cytotoxic effect of the studied compounds on A2058 and MeWo cells. (**A**–**B**) Dose and time-dependent effect of compounds **2**, **4**, and **5** on the viability of (**A**) A2058 and (**B**) MeWo cells. Cells were treated with the concentrations indicated during 4.5, 8, and 12 h. Following treatment either XTT or Presto Blue were added, and samples were measured in a spectrophotometer after 4 or 2 h, respectively. The values are expressed as means ± SD obtained from three independent experiments.

A2058 and MeWo cells were treated with increasing concentrations of compounds **1–6** and cell viability assays with XTT or Presto Blue were carried out at different incubation times. Cell viability decreased with compounds **1**, **2**, **4**, and **5** (Figure 4), whereas no toxicity was observed for compounds **3** and **6** even at concentrations that exceeded four times the IC_50_ values estimated with the isolated chaperone system). Compound **1** had the highest inhibitory effect but it was discarded from the study based on published work that reported its toxicity in several non-transformed cell lines [52,53,54]. Compounds **2** and **5** showed an expected time-dependent toxicity, with a general difference in their effectiveness between the two melanoma cell lines, being more toxic for A2058 as compared to MeWo cells (Figure 4). With compound **4**, a decreased potency over time was observed (Figure 4), which could be attributed to compound chemical instability, compound efflux or drug inactivation, among others [55].

The viability results were confirmed by cell culture images (Figure S4), which showed increased cell shrinkage and membrane blebbing, indicative of cell death, when compared to non-treated cells [56]. Images of treated cell cultures were not taken at longer incubation times because the cells detached from the flask surface upon cell death.

### 2.5. Melanoma Cell-Specific Inhibition by the Selected Compounds

**Figure 5:**
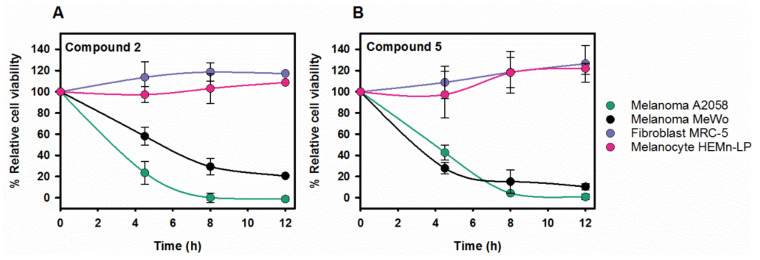
The selected compounds do not compromise the viability of melanocytes and fibroblasts whereas they are cytotoxic on melanoma cell lines. Time-dependence of the viability of the four cell lines after treatment with compounds **2** (40 µM) (**A**) and **5** (20 µM) (**B**). While melanoma cell viability decreased with time, neither fibroblasts nor melanocytes reduced their viability after 12 h. Following treatment, either XTT or Presto Blue were added, and the absorbance of samples was measured in a spectrophotometer after 4 or 2 h, respectively. Values are expressed as mean ± SD obtained from three independent experiments; 100% viability refers to each cell line without compound. Lines in panels A and B are to guide the eye.

Once confirmed that compounds **2** and **5** were toxic for the melanoma cell lines A2058 and MeWo, it was necessary to address whether their effect was specific for cancer cells. Based on the initial hypothesis, transformed cells rely to a greater extent on Hsps than normal cells do and, hence, inhibition of the target proteins would be more detrimental to the former. To find out if this was the case for these compounds, two human control cell lines were selected: melanocytes (HEMn-LP non-malignant skin cells) and fibroblasts (MRC-5). Melanocytes were primary culture cells from skin and fibroblasts immortalized cells. Control cell lines were treated with compounds **2** (40 µM) and **5** (20 µM), and no toxicity was observed in either case. Thus, melanocytes and fibroblasts were resistant to these molecules while they caused cell death in A2058 and MeWo cells in the conditions tested (Figure 5A,B).

Toxicity of compound **5** with the melanoma cell lines was observed at around 7-fold lower concentrations than the IC_50_ values determined for the isolated chaperone system, suggesting that this compound could have additional targets (see discussion). As this makes it considerably more difficult to characterize the action of the potential drug and control the emergence of side effects, compound **5** was omitted from the study at this stage and research continued with compound **2** (Pinaverium bromide; PB).

### 2.6. Pinaverium Bromide (PB) Causes Melanoma Cell Death by Inducing Apoptosis

**Figure 6:**
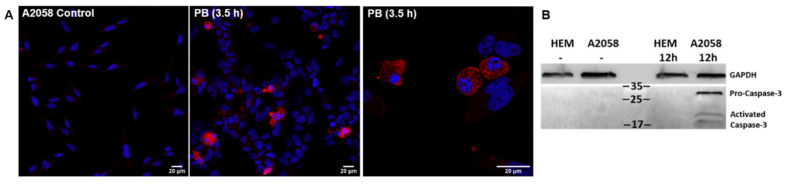
Detection of activated caspase-3 in A2058 cells as an indicator of apoptosis. (**A**) A2058 cells in the absence (control panel) and presence of PB (40 µM). Samples were stained for activated caspase-3 (red) as an indicator of apoptosis and cell nuclei (blue) were detected with 4′,6-diamidino-2-phenylindole. (**B**) Immunoblot of caspase-3 corresponding to human melanocytes (HEM) and A2058 cells treated 12 h with 20 μM PB. GAPDH is used as loading control.

To explore whether PB could lead to melanoma cell apoptosis, we performed immunofluorescence assays to detect activated caspase-3, one of the apoptosis effector/executioner caspases [57]. Results showed that 40 µM PB induced caspase-3-dependent apoptosis after 3.5- and 7.5-h treatment in A2058 (Figure 6A) and MeWo cells (Figure S5), respectively. A shorter incubation time was used with A2058 cells due to its higher sensitivity to PB causing loss of viability and cell detachment from the culture flask over time. Additionally, melanoma cell cultures treated with 40 µM PB were recorded for 8 h (A2058) and 24 h (MeWo) to monitor compound-induced morphological changes prior to cell death (Figure S6). Detection of active caspase-3 in A2058 cells but not in melanocytes upon exposure to a toxic PB concentration further reinforces apoptosis as the main cause of cell death (Figure 6B and Figure S7).

### 2.7. Identification of PB Binding Site by Docking and Molecular Dynamics

We resorted to molecular modeling to identify the potential binding site of PB in its targets, and to describe the network of protein–ligand interactions that could stabilize the complexes. To this aim, we first performed blind protein–ligand docking experiments with GaudiMM [58] to assess the most likely binding site of the drug, and afterwards the most accurate orientation was generated with GOLD [59]. Finally, large-scale classical molecular dynamics (MD) was performed on the best solutions of the docking experiments to explore how compound binding affected protein motions.

As PB bound to Apg2 and to the ADP- and ATP-states of Hsc70, it was reasonable to suggest that this drug should interact with a region shared by these proteins. Therefore, docking solutions were filtered focusing on regions that appear in these structures. The initial blind docking performed with GaudiMM allowed us to discriminate two potential binding pockets (Figure S8). The first site, which had the higher density of docking solutions, showed interactions with the NBD domain and the linker (Figure S8A). The second site involved only residues located on the NBD domain, mainly at the external region of the IA subdomain (Figure S8A).

**Figure 7:**
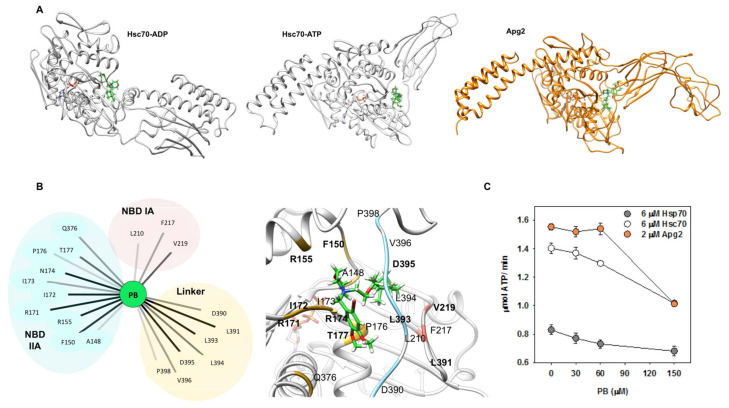
PB binds to Hsc70 and Apg2 through interactions with the NBD and the linker and impairs their ATPase activity. (**A**) Best docking results of PB (green) with Hsc70 in ADP and ATP states, and with Apg2. (**B**) Interactions between PB (green) and Hsc70-ATP (light grey) that allow the compound to be stable in the initial predicted binding pocket after a MD simulation of 100 ns. Interacting residues are grouped and colored depending on their location in the three-dimensional structure of the protein: IA subdomain of the NBD in pink, NBD IIA subdomain in yellow and the linker in blue. (Left panel) Interaction network between PB and Hsc70 in ATP state performed after analyzing the MD simulation of this complex using Cytoscape. Bold lines correspond to the most frequent and thus, the most stabilizing interactions. (Right panel) Representative frame of the PB-Hsc70 complex during the simulation. (**C**) Effect of PB on the ATPase activity of Apg2, Hsc70, and Hsp70. Increasing concentrations of the compound were added to 6 µM Hsc70 (white), 6 µM Hsp70 (grey), or 2 µM Apg2 (orange). The ATPase activity of the chaperones showed a concentration-dependent inhibition in the presence of PB. Values are expressed as means ± SD obtained from three independent experiments.

When a more detailed docking to these protein regions was carried out with GOLD, PB showed better binding to the first site for Apg2, Hsc70-ADP, and Hsc70-ATP, although the difference was stronger for the Hsc70-ATP state. Based on these results, we considered this site at the region connecting the NBD and SBD subdomains (Figure 7B) the most probable location for PB. We next performed MD simulations (100 ns) on the best docking solution, the PB/Hsc70-ATP complex, to explore its stability and to identify the residues involved in complex formation. As the ligand remained in the same location, with only minor changes in orientation, the interaction of the compound with the protein was statistically analyzed with Cytoscape [60] as implemented in UCSF Chimera [61] (Figure 7B). Results showed that residues from subdomains IA and IIA of the NBD (L210, F217, and V219 and A148, F150, R155, R171, I172, I173, N174, P176, T177, and Q376, respectively) and from the linker (D390, L391, L393, L394, D395, V396, and P398) were involved in complex formation. They also suggested that binding is dominated by Van der Waal interactions, and thus that complex stabilization largely depends on hydrophobic contacts (Figure 7B). These findings are consistent with the high hydrophobicity of both the ligand and the binding site. Docking also suggested the same binding site in Hsp70 (Figure S8B). As expected, sequence alignment showed that the amino acids engaged in PB binding are conserved in the three proteins (Figure S8C).

The involvement of these protein regions in complex formation indicates that PB binding might affect the ATPase activity of these proteins. To test this possibility, we analyzed the ATP-hydrolysis rate of Hsc70, Hsp70, and Apg2 at increasing compound concentrations (Figure 7C). The results showed that PB inhibited 23–28% and 35% of the ATPase activity of Hsp70-Hsc70 and Apg2, respectively, in a concentration-dependent manner.

### 2.8. PB Inhibits Luciferase Protein Refolding in a Cellular Context

**Figure 8:**
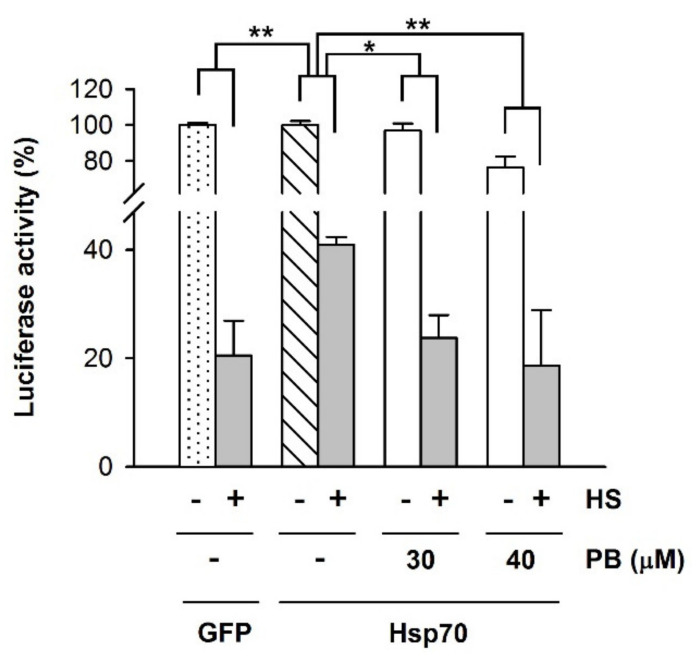
PB inhibits Hsp70-mediated refolding of luciferase in HEK-293 cells. Luciferase-encoding plasmids were co-transfected with plasmids encoding Hsp70 or GFP. Cells were subjected to heat shock (HS), 45 °C during 30 min, and after a recovery period of 2 h at 37 °C in the absence or presence of PB (30 and 40 μM) as indicated, luciferase activity was measured. Values for samples co-expressing Hsp70 are expressed relative to that obtained for the unheated control in the absence of PB (white striped column). For cells co-expressing GFP, 100% was the corresponding control without HS (white dotted column). Statistical analysis of the heated samples (grey columns) was performed considering as 100% the luciferase activity obtained without thermal stress for each experimental condition (white columns). Data are presented as mean ± SD of three independent experiments (* *p* < 0.05; ** *p* < 0.01).

To explore if PB inhibited the activity of the Hsp70 system in a cellular context, we resorted to study its effect on the chaperone-mediated substrate protein recovery after heat shock. To this aim, Hsp70 (HspA1A) was used, instead of Hsc70 (HspA8), as it was found to be significantly more efficient to refold luciferase after heat shock, in agreement with a previous study [62]. HEK-293 cells co-expressing luciferase and Hsp70 or GFP as a control were subjected to heat shock conditions (45 °C, 30 min), and the recovery of luciferase activity was monitored after the thermal stress in the absence and presence of two PB concentrations toxic for melanoma cell lines (Figure 8). Hsp70 showed a high activity on luciferase refolding compared to control cells co-expressing GFP (Figure 8A), as previously reported [62]. Interestingly, PB concentrations that induced death of the two melanoma cell lines without affecting the viability of non-tumor cells (Figure 5), including HEK-293, inhibited luciferase recovery, which became similar to that of the control sample expressing GFP instead of Hsp70 (Figure 8). This is clearly observed at 30 μM PB, compound concentration that did not significantly change the amount of active luciferase before the heat shock. At 40 μM PB, the 28% inhibition observed under non-stress conditions is lower than the 58% decrease estimated after thermal stress (Figure 8).

Taken together, our results reveal that PB inhibits the intracellular chaperone activity of the Hsp70 system and induces apoptosis in the tumorigenic melanoma cell lines A2058 and MeWo but not in non-malignant skin cells, melanocytes, and fibroblasts.

## 3. Discussion

We first aimed to identify compounds that could specifically target Apg2, a member of the Hsp110 family, without interacting with Hsc70. The peculiarities of Apg2 compared to Hsp70, namely an insertion in the SBD and an extension at its C-terminus [49], were considered appealing enough to try to find modulators that would bind to the mentioned regions and, hence, be specific for Apg2. However, the six compounds selected as hits for analysis after the screening and validation of the Apg2 modulators do not interact with these Apg2-specific IDRs. This is supported by the finding that they bind to deletion variants of the protein that lack the specific regions that differentiate it from Hsc70. This is not surprising, as it has been proven difficult to identify or design small chemical molecules that specifically bind to intrinsically disordered proteins (IDPs) [63,64]. Thus, it was not unexpected that these compounds also interacted with Hsc70 as revealed by DSF and by their inhibitory effect on the refolding activity of both the ternary (Hsc70/DnaJB1/Apg2) and binary (Hsc70/DnaJB1) chaperone complexes. Therefore, they most probably bind to structural regions shared by the two proteins, as suggested by ATPase, molecular docking and MD studies, being Hsp110 binders also capable of associating to Hsc70 and Hsp70. Among the distinct Hsp families, Hsp70 is considered the hub that connects the different branches involved in maintaining protein homeostasis (proteostasis): folding, trafficking and degradation [65]. Hsp70 has been implicated in the bad prognosis of cancer as its overexpression has been linked to anticancer drug resistance, inhibition of cell apoptosis and increasing survival rates under otherwise adverse conditions [30,66,67].

Melanoma is the most aggressive form among the different skin cancers [68]. The incidence and mortality rate of malignant melanoma has been increasing worldwide. Its treatment mainly depends on the time of diagnosis, as it becomes refractory to conventional therapies in the metastatic stage [69]. Therefore, it is necessary to develop new drugs, which by themselves or in combination with existing medications can overcome the resistance against chemotherapy and induce cell death, an approach that includes the possible repositioning of old drugs for new treatments. Drug repositioning or repurposing aims to find new therapeutic uses to approved drugs beyond the original medical indication they were given [70]. Furthermore, this field has allowed the development of polypharmacology, which is based on the fact that a certain molecule can target several targets associated to multifactorial diseases [71].

Several studies have shown that Hsp110 and Hsp70 are overexpressed in melanoma, as in other cancers [72,73], playing a key role in cancer progression/prognosis and drug resistance [74]. Cancer cells with higher metabolic needs and hyperactivated signaling pathways, exhibit a stronger demand for chaperone machineries to survive [15]. It has been suggested that in melanoma cells, their tumorigenic potential seems to correlate with the ability to disable apoptosis, a process in which Hsp70 is a key regulator [74,75]. Hsp70 binds and inactivates a number of pro-apoptotic molecules, i.e., caspase 3/7, and thereby delays cell death [22,76,77]. Elevated chaperone levels block the apoptotic pathway at different stages. Therefore, Hsp70 inhibitors show tumor-selective cytotoxicity, with limited toxicity in normal cells [74]. Hsp70 is an obligate co-chaperone for Hsp90, and targeting the latter has shown a key role in the treatment of melanoma [78]. It has been hypothesized that combining Hsp70 and Hsp90 inhibitors might lead to enhanced activity [30,78,79]. However, interestingly, it has also been demonstrated that inhibition of just Hsp70 considerably modifies and severely damages the Hsp90 chaperone system, pointing to Hsp70 as a new therapeutic objective for melanoma [30,79].

Cell viability experiments showed that among the six compounds that bind Apg2 and Hsc70, only PB induced cell death at concentrations close to those described in the reactivation assays and inhibits the intracellular chaperone activity of the Hsp70 system, thus suggesting that inhibition of this chaperone system might be behind the observed toxic effect. As desired, toxicity was only detected in the two cancer cell lines and not in fibroblast or melanocytes, indicating that it could be related to their strict chaperone-dependent proliferation. Inhibition of the Hsp70 system was therefore expected to cause cell death in the metabolically demanding melanoma cell lines. This compound, as suggested by molecular docking and MD simulations, interacts with a conserved chaperone surface made of residues from subdomains IA and IIA of the NBD and the linker. This binding mode could explain the effect of the compound on both members of the Hsp70 family and Apg2, and the inhibitory effect on the complete Hsp70 chaperone system. Loss of cell viability at compound **5** (Mefloquine Hydrochloride, MH) concentrations around seven-fold lower than those observed in the aggregate refolding assays, most likely indicates that the effect of this drug could be due to a mechanism different from chaperone inhibition. Indeed, it has been reported that similar MH concentrations exert antiproliferative effects through an increased lysosomal biogenesis and activation, followed by oxidative stress, lysosomal lipid damage and functional impairment [80].

This work suggests that PB could be repurposed as an inhibitor of Hsp70 for melanoma treatment. This compound is a spasmolytic agent used for functional gastrointestinal disorders. PB action restores normal bowel function, relieving spasm and pain, transit disturbances and other symptoms related to motility disorders and may be considered as effective first-line therapy for patients with irritable bowel syndrome [81]. It is known that PB functions as an atypical calcium antagonist that interacts with the 1,4-dihydropyridine binding sites on voltage dependent L-type calcium channels, located on gastro intestinal smooth muscle cells, in a competitive manner [82]. Based on this mechanism of action and on the involvement of calcium channels in cell cycle progression, it has been proposed that existing calcium channel blockers may be used in combined therapies against melanoma [83]. Our results show that this compound also inhibits the intracellular chaperone activity of the Hsp70 system, and thus point to Hsp70 as an alternative target of PB that could be, at least in part, responsible for its marked toxicity against two melanoma cell lines without compromising the viability of fibroblast and primary melanocytes. The around two-fold difference in the IC_50_ values estimated for PB in cell death assays (around 10–33 μM for A2058 and MeWo melanoma cell lines, respectively; Figure 4) and in vitro reactivation experiments (55–70 μM; Table 2), could be due to its possible action on different targets, such as calcium channels (see below) and the Hsp70 system, which might exert a synergistic effect that would result in an increase cell toxicity [71]. This has been considered a potential advantage of drug repurposing, as a drug active on multiple targets may be characterized by an improved efficacy when compared to a more selective pharmacological agent.

The comparison of the PB-induced toxicity in the two melanoma cell lines also suggests that A2058, which has a doubling time significantly shorter than that of MeWo, 30 and 40 h, respectively, is more sensitive to the compound. This result is expected, as the accelerated metabolism needed to proliferate faster depends to a higher extent on the chaperone machinery [29]. A2058 cells, in contrast to MeWo, harbor a point mutation (V600E) in the BRAF gene [84] that is considered a proto-oncogene coding for a serine/threonine kinase. In line with the doubling time differences between A2058 and MeWo cells, mutations in BRAF are related to an accelerated proliferation [85]. PB induces cell death by activating apoptosis in the two melanoma cell lines as determined by the detection of active caspase-3 in treated-cells [57]. Blebbing, another apoptotic hallmark, was identified in both A2058 and MeWo cells, as determined by time-lapse cell culture analysis.

It is now generally accepted that Hsps play a pivotal role in tumor progression and resistance to current therapies and thus, they appear as potential targets in the development of new cancer treatments [72]. This study has identified an inhibitor of the human Hsp70 system and is capable of inducing cell death via apoptosis in the melanoma cell lines A2058 and MeWo without affecting the viability of melanocytes and fibroblasts. It remains to be explored whether PB could be used as a monotherapy without the undesired toxicity shown by other Hsp70 and Hsp90 inhibitors that limits their clinical efficacy [86], or, alternatively, it could be administered in combination with standard chemotherapeutic agents [87] or other Hsp inhibitors [29] to enhance their antitumor potency.

## 4. Materials and Methods

### 4.1. Cloning, Expression and Purification of Proteins

The cDNAs of Apg2 (HSPH2), Hsc70 (HSPA8), and DNAJB1 were obtained from Addgene and cloned into pE-SUMO vector (LifeSensors, Malvern, USA). The mutants Apg2ΔAS and Apg2ΔC, carrying a deletion from residue 504 to 569 and 702 to 840, respectively, were cloned by fusing two PCR fragments corresponding to the upstream and downstream sequences of these protein segments [88] and verified by sequencing. Recombinant proteins containing a tag with 6 histidines and SUMO fused to the N-terminus were expressed in BL21 Rosetta cells and purified with a first step of affinity chromatography using NiNTA (Qiagen, Hilden, Germany) columns, followed by treatment with His-Ulp1 to cleave the tag, and a final NiNTA column in which the pure protein eluted in the unbound fraction [89].

### 4.2. Differential Scanning Fluorimetry (DSF)

High-throughput screening was carried out using DSF with 0.1 mg/mL Apg2, Apg2 ΔL or Apg2 ΔC in 25 mM Hepes pH 7.6, 2, 0.01% TX-100, 2 mM DTT and 5x Sypro Orange dye (Sigma-Aldrich, St. Louis, MI, USA). Protein samples (9.6 µL) were placed in 384-well PCR plates (Thermo Fischer Scientific, Waltham, MA, USA) and compounds from the Prestwick Chemical Library^®^ (1280 compounds dissolved in DMSO at a concentration of 10 mM) were added to a final concentration of 400 µM and 4% DMSO. Each plate had 16 control wells with 4% DMSO but without compounds. Plate preparation was done using the Bravo Automated Liquid Handling Platform (Agilent, Santa Clara, CA, USA). Apg2 validated binders were similarly tested with Hsc70. In this case, samples contained 0.1 mg/mL protein in 40 mM Hepes pH 7.6, 50 mM KCl, 5 mM MgCl_2_, 0.01% TX-100, 2 mM DTT and 5 × Sypro Orange in the absence or in the presence of 2 mM ADP or 2 mM ATP. Plates were loaded into a RT-PCR Light Cycler 480 II (Roche, Basel, Switzerland). Thermal unfolding curves were recorded from 30 to 90 °C at 2 °C/min scan rate by following the Sypro Orange fluorescence intensity (λ_ex_ = 465 nm, λ_em_ = 580 nm) associated with protein unfolding. Validation of the selected hits was carried out by testing at least eight different compound concentrations within the concentration range 0.195–400 µM in a final volume of 12.5 µL using 96-well PCR plates (Thermo Fischer Scientific). Sample buffer and assay conditions were the same as above.

Analysis of the experimental unfolding curves was carried out as reported [90]. Fluorescence data were smoothed and the first derivative of the thermal unfolding curves were calculated. The *T*_m_-values were obtained as the maxima of the first derivative of the experimental unfolding curves. Compounds that altered the *T*_m_ value ≥ 3-fold × standard deviation (SD) of the *T*_m_ for DMSO controls (*T*_m0_) were considered potential hits. The concentration dependent *T*_m_-values were fitted to Equation (2) in Cooper and McAuley-Hecht [41].

### 4.3. G6PDH Aggregate Reactivation

G6PDH (2.5 µM) was heat-denatured and aggregated for 30 min at 50 °C in 50 mM Tris-HCl pH 7.5, 150 mM KCl, 20 mM MgCl_2_, and 10 mM DTT. Subsequently, aggregates were stabilized for 30 min at 30 °C. The properties of these aggregates have been previously reported, and typically show a hydrodynamic radius of approximately 500–600 nm [91]. Aggregates (0.4 µM final concentration) were added to samples containing 2 µM Hsc70 and 0.5 µM DnaJB1 in the absence or presence of 0.4 µM Apg2. The assay buffer also contained 0.01% TX-100 and 1–2% DMSO to avoid self-association of the compounds and maximize compound solubility. A preincubation of the samples with compounds for 30 min at 30 °C was done prior to starting the reaction. The reaction was started with the addition of 2 mM ATP and the ATP-regeneration system (3 mM PEP and 20 ng/mL PK). Final sample volume was 100 µL. Reactivation was monitored at 30 °C for 180 min. At different times of the reaction, 5 µL of sample were added to 250 µL buffer containing 50 mM Tris-HCl pH 7.5, 50 mM KCl, 20 mM MgCl_2_, 10 mM DTT, 1 mM NADP^+^, 2.5 mM G6P (Sigma-Aldrich). NADPH formation was measured recording the absorbance increase at 340 nm in a Synergy HTX plate reader (BioTek, Winooski, VT, USA) in transparent 96-well plates with flat bottom (Sarstedt, Newton, NC, USA) for 5 min. Reactivation percentages were calculated considering the slope of NADPH formation for native G6PDH as 100% activity. Aggregates not treated with chaperone gave the spontaneous reactivation (below 5%) that was established as 0% reactivation. IC_50_ values were estimated with a sigmoidal dose-response ligand-binding equation.

### 4.4. ATPase Measurements

Steady-state ATPase activity was measured using the assay described by Nørby [92]. Briefly, Assays were performed at 30 °C in 40 mM Hepes, pH 7.6, 50 mM KCl, 5 mM Mg acetate, and 1 mM ATP, using transparent 96-well plates with flat bottom (Sarstedt), a final volume of 200 µL and 6 µM Hsc70 or 2 µM Apg2. Assay buffer also contained 0.01% Triton X-100 (TX-100) and 1–2% dimethyl sulfoxide (DMSO). ATP consumption rates (µmol ATP/min) were calculated from the slopes of the A_340_ decay curves over the selected time intervals that showed a linear absorbance decline. The amount of ATP moles hydrolyzed per min was calculated measuring the absorbance decay at 340 nm during 1 h in a Synergy HTX plate reader (Biotec, Emmerich am Rhein, Germany), using the extinction coefficient of NADH (ε_340_ 6220 M^−1^ cm^−1^).

### 4.5. Cell Culture

Tumorigenic skin melanoma cell lines A2058 (ATTC^®^ CRL-11147™) and MeWo (ATTC^®^ HTB-65™) were obtained from the American Type Culture Collection (Rockville, MD, USA). Cells grew in Dulbecco’s Modified Eagle Medium (DMEM) high glucose (Sigma-Aldrich) supplemented with heat-inactivated 10% fetal bovine serum (FBS) (HyClone™, GE Healthcare, IL, USA). Lung MRC-5 fibroblasts (ATTC^®^ CCL-171™) from the American Type Culture Collection (Rockville, USA) were grown in Roswell Park Memorial Institute (RPMI) Medium 1640 + GlutaMAX™ (Gibco™, Life Technologies, Carlsbad, CA, USA) supplemented with heat-inactivated 10% FBS. Primary human melanocytes (human epidermal melanocytes, neonatal, light pigmented, HEMn-LP) were purchased from Invitrogen (Waltham, MA, USA, C-002-5C). Cells were grown in Cascade Biologics Medium 254 (Gibco™, Life Technologies) supplemented with Cascade Biologics HMGS-2 (Gibco™, Life Technologies).

All cell lines were grown at 37 °C in a 5% CO_2_-95% air-water saturated atmosphere in 25 or 75 cm^2^ flasks as exponentially growing subconfluent monolayers. Cells were examined during culture with an inverted microscope Olympus CK-2 (Olympus, Waltham, MA, USA) and regularly passaged at subconfluency. Prior to detachment, cells were washed with PBS and treated with 0.05% trypsin-EDTA (Gibco™, Life Technologies) for 5 min at 37 °C. Trypsin was inactivated by the addition of FBS (except for primary melanocytes) and diluted twice by adding new fresh growth medium. Cells were centrifuged at 4 °C and 300× *g* for 5 min, and the resulting pellets were resuspended and quantified in a TC20™ Automated Cell Counter (Bio-Rad, Madrid, Spain) with trypan blue staining. Cells were plated at 1:2–1:10 dilutions in a final volume of 10 mL (75 cm^2^ flasks) or 5 mL (25 cm^2^ flasks) in the corresponding growth medium.

### 4.6. Recovery of Intracellular Luciferase Activity

pcDNA5/FRT/TO HSPA1A (Addgene) was used to express the human inducible Hsp70, pRSVLL/V [93] encoding for cytoplasmic firefly luciferase (cyt-luciferase) was kindly provided by Harm H. Kampinga, University of Groningen [94], and pcDNA5/FRT/TO GFP (Addgene) was used as a control. HEK (human embryonic kidney)-293 cells were grown in Dulbecco’s Modified Eagle Medium (DMEM) supplemented with 10% FBS (Gibco). Cells (2.0 × 10^5^) were seeded in an 8.87-cm^2^ culture dish (Sarstedt). After 24 h, they were transfected with 1.5 μg pRSVLL/V with Lipofectamine^®^ 2000, following the DNA Transfection Reagent Protocol (Invitrogen). When required, cells were cotransfected with pcDNA5 HSPA1A (1.5 μg) or with pcDNA5 GFP so that in all experiments the total plasmid quantity was 3 μg/8.87-cm^2^ dish. Moreover, 48 h after transfection cells were subjected to heat shock (45 °C, 30 min in a water bath). The medium was replaced by DMEM supplemented with 1% FBS containing cycloheximide (20 μg/mL) 30 min before the heat shock. After heat shock, 30 or 40 μM of PB was added to the corresponding samples and 0.9% DMSO to the controls, and the plates were placed in a CO_2_ incubator at 37 °C for 2 h. Afterwards, cells were washed with ice-cold phosphate-buffered saline (PBS), lysed in 300 μL of luciferase lysis buffer (25 mM H_3_PO_4_/Tris, pH 7.8, 10 mM MgCl_2_, 1% Triton X-100, 15% glycerol, 1 mM ethylenediaminetetraacetic acid) containing 0.5% 2-mercaptoethanol, and luciferase activity was measured in a Synergy HTX plate reader (Biotec) after the addition of 50 μL of Luciferase Assay Reagent (Promega, Southampton, UK) to 100 μL of sample. The activity of samples that were treated in the same way, but with the heating step replaced by a 30 min incubation on ice, was taken as 100% for each condition, as previously reported [62].

### 4.7. Immunocytochemical Staining

Immunofluorescence was used to detect activated caspase-3 in A2058 and MeWo cells treated with 40 µM PB; 40,000 cells/well of A2058 or MeWo were plated in poly L-lysine-coated 8-well chambered coverslips (µ-Slide 8 well, Ibidi, Grafelfing, Germany) in 300 µL DMEM supplemented with 10% FBS and incubated overnight at 37 °C. The following day, the medium was replaced by 300 µL DMEM supplemented with 1%FBS and 0.9% DMSO in the presence or absence (controls) of 40 µM PB. A2058 and MeWo cells were incubated for 3.5 or 7.5 h, respectively. The two melanoma cell lines were not subjected to the same incubation times due to their different sensitivity to PB. In both cases, the incubation time aimed at obtaining samples where death occurred without cell detachment. After the incubation, the medium was removed and cells were washed with PBS. Cell fixation and permeabilization was carried out with 70% cold methanol for 30 min at −20 °C, and the chambers were washed with 1% PBS. Cells were rinsed three times with 1% BSA and 0.02% sodium azide in PBS and blocked for 30 min at room temperature with the same solution. The primary antibody to detect activated casapase-3 (rabbit polyclonal to cleaved caspase-3 (Asp175), (Cell Signaling Technology, Danvers, MA, USA) was dissolved in 0.5% BSA and 0.1% sodium azide in PBS and added to the chambers as 1:400 dilution. Cells were incubated with primary antibodies overnight at 4 °C. The next day, chambers were washed with 1% BSA and 0.02% sodium azide in PBS three times before addition of the secondary antibody. Cells were incubated with goat anti-rabbit Alexa Fluor 594 (Invitrogen) diluted at 1:200 in PBS supplemented with 0.5% BSA and 0.1% sodium azide for 45 min at room temperature. After three rinses with 1% BSA and 0.02% sodium azide in PBS, cells were incubated with 4,6-diamidino-2-phenylindole (DAPI) (Sigma-Aldrich) for 15 min at room temperature at a concentration of 1 µg/mL in PBS to stain the nuclei. Lastly, cells were rinsed with PBS twice, kept in the same solution at 4 °C and viewed with a confocal microscope LSM 800 (Zeiss, Birmingham, UK) using a Plan Apochromat 20× NA 0.8 objective (Zeiss).

### 4.8. Western Blot Analysis

Western Blot was used to detect activated caspase-3 in controls and in A2058 melanoma cells and primary melanocytes treated with 20 μM PB; 10^6^ cells/well of A2058 and melanocytes were plated in 6-well microplate in 2 mL DMEM supplemented with 10% FBS and incubated overnight at 37 °C. The following day, the medium was replaced by 2 mL DMEM supplemented with 1% FBS and 0.9% DMSO in the absence (controls) and presence of 20 μM PB and A2058 and primary melanocytes were incubated for 12 h. Cells were lysed in RIPA buffer supplemented with protease inhibitors. The total protein concentrations were measured by DC protein assay (Bio-Rad), and equal amounts of proteins (25 µg) were resolved by SDS PAGE and transferred to a nitrocellulose membrane (Life Technology, Alcobendas, Spain). Blots were incubated with PBS containing 5% Bovine Serum Albumin and 0.1% Tween-20 for 1 h to block non-specific binding. Proteins were probed with cleaved caspase-3 (Cell Signaling Technology) rabbit monoclonal specific antibody (dilution 1:1000) to detect activated caspase-3, followed by an anti-rabbit secondary antibody (Invitrogen) (dilution 1:1000) conjugated with HRP. After mild stripping, the membrane was analyzed with mouse monoclonal GAPDH specific antibody (Abcam, Cambridge, UK) (dilution 1:1000), followed by an anti-mouse secondary antibody (Cell Signaling Technology) (dilution 1:1000) conjugated with HRP. The HRP signals were developed by using by enhanced chemiluminescence using the SuperSignal^®^ West Pico Chemiluminescent Substrate (Thermo Scientific, Rockford, IL, USA).

### 4.9. Viability Assays

Two colorimetric assays were used to study the cytotoxic effects of the selected compounds: the XTT Cell Proliferation Kit II (Roche) and Presto Blue™ Cell Viability Reagent (Invitrogen). Exponentially growing cell lines seeded at 20,000 cells/well in 96-well flat bottom plates (Sarstedt) were incubated overnight in 150 µL of the corresponding growth medium. The following day, the medium was replaced by fresh medium (150 µL) containing 1% FBS (except for melanocytes) and 0.9% DMSO in the presence or absence of different concentrations of compounds. A2058 and MeWo cells were treated with increasing compound concentrations for cell viability assays. In the case of primary melanocytes and fibroblasts, one concentration was tested for each compound: 40 µM compound **2**, 40 µM compound **4**, and 20 µM compound **5**. Cells were incubated for 4.5, 8, and 12 h with subsequent addition of XTT (50 µL) or Presto Blue (17 µL) to each well followed by an incubation for 4 or 2 h, respectively. Cell viability in plates treated with XTT was determined by reading the absorbance at 490 nm in a Synergy HTX plate reader (BioTek). Viability in plates treated with Presto Blue was determined in the same manner but results were obtained by subtracting the absorbance at 570 nm to that obtained at 620 nm. In both cases, the absorbance of the medium was subtracted from cell-containing wells. Control values (without compounds but with 0.9% DMSO) were taken as 100% viability.

### 4.10. Time-Lapse Imaging

A2058 and MeWo cell lines were treated with 40 µM PB, for time-lapse live cell imaging to analyze the morphological changes they underwent prior to cell death. Moreover, 300,000 A2058 and MeWO melanoma cells were seeded in culture medium (2 mL DMEM supplemented with 10% FBS) in a 35 mm glass bottom dish (µ-Dish 35 mm, Ibidi) and allowed to attach overnight. Cells were incubated in 2 mL DMEM supplemented with 1% FBS, 0.9% DMSO and 40 µM PB. This culture was recorded by taking photos at a 20× magnification (10 different frames per dish) every 10 min during 8 (A2058) or 24 h (MeWO) with the BioStation IMQ live-cell time-lapse microscopy (Nikon Instruments B.V.) at 37 °C and 5% CO_2_.

### 4.11. Homology Modeling

The first step to generate the homology models was to search for the best templates using the SWISS-MODEL web server [95]. Two models (ADP and ATP state) were generated for Hsc70 and Hsp70, as these states adopt different structures. The ADP state of Hsp70 was generated using PDB ID 2KHO.A as template [96], and its ATP state with PDB ID 6ASY.A [97]. The ADP state of Hsc70 was modeled using PDB IDs 1YUW.A [98] and PDBID 2KHO.A [96] as templates, and its ATP state using PDB IDs 5E84.A [99] and 1KAX.A [100]. The template used for Apg2 was PDB ID 3C7N.A [101]. Then, the templates were aligned with the sequences of the query proteins considering the structure. Those structure-based alignments were employed to generate models using MODELLER [102]. Different models were obtained for each protein, and all of them were analyzed by ERRAT [103], Verify-3D [104], and WHAT-IF [105] to assess their quality. Finally, the best models were energy minimized using OMMProtocol [106], achieving good quality initial homology models.

### 4.12. Protein–Ligand Docking

A first docking round was performed to search for the most probable binding sites for the ligand using GaudiMM [58]. These results, considering the DSF results, were used to select the most suitable binding regions for a more detailed docking study using GOLD5.8 [59], and evaluated with the ChemScore scoring function [107]. Rotamers for residues occupying the active site were applied using the Dunbrack library [108].

### 4.13. Molecular Dynamics Simulations

Molecular Dynamics (MD) simulations of the best docking result (Hsc70-ATP state) were performed to ensure the stability of the ligand-protein complex and to potentially refine the initial protein–ligand docking through the exploration of the conformational space. The complex was prepared for the all-atoms MD simulations: the histidines predicted to be in a region of higher pH than 6.0 by the H++ server [109] were protonated in the initial models and the system was set up with XLeap [110]. Explicit solvent was used for initial simulations, and Na+ ions were added to obtain charge neutrality. The model system was embedded into a cubic box with a distance of 6 Å between the protein and the box edge, containing around 60,000 water molecules.

AMBER ff99SBildn [111] and TIP3P [112] force fields were used for proteins and water, respectively, and the parameters for the ions used to neutralize the system were taken from ions94.lib library. Parameters for ATP, Mg^2+^ and K^+^ were not included in AMBER18 by default, so they were taken from the literature [113,114]. In the case of the PB ligand, parameters were developed according to standard approaches. Point charges were calculated with antechamber [115] according to the RESP procedure [116] and GAFF force field was adopted for the remaining atoms [117].

MD simulations were performed with OpenMM [118]. A cutoff of 1 nm was used for short-range electrostatics and Van der Waals interactions. Long-range electrostatic interactions were calculated with the particle mesh Ewald (PME) method, using periodic boundary conditions [119]. Bonds involving hydrogen atoms were constrained using the SHAKE algorithm [120]. A time step of 1 fs was used to integrate the equation of motion with a Langevin integrator [121]. Constant temperature and pressure were achieved by coupling the systems to a Monte Carlo barostat at 1.01325 bar [122]. Model system was energy minimized before starting the MD simulations, allowing movement of water molecules, sidechains, and backbone atoms. Then, temperature was increased from 100 K to 300 K and 100 ns MD simulations were carried out. Molecular graphics were made with the UCSF Chimera package [61].

### 4.14. Statistical Analysis

All measurements were performed at least three times and levels of significance were determined by a two-tailed Student’s *t*-test. A value of *p* < 0.05 was considered statistically significant (* *p* < 0.05; ** *p* < 0.01).

## 5. Conclusions

The findings from this study show that pinaverium bromide inhibits the intracellular activity of the Hsp70 system, which could be one of its targets, inducing specifically the death of two melanoma cell lines without being cytotoxic for non-tumorigenic cells. They support further clinical investigations in mouse melanoma models to explore the possibility of adding this compound to the therapeutic arsenal for melanoma treatment.

## Figures and Tables

**Table 2:** IC_50_ values estimated for the inhibition of the aggregate reactivation ability of binary and ternary chaperones mixtures by the selected compounds. Values are means ± SD of three independent experiments.

Compound	IC_50_ (µM)	
	G6PDH Reactivation	
	Hsc70 + DnaJB1	Hsc70 + DnaJB1 + Apg2
**1**	11.9 ± 0.2	14.5 ± 1.8
**2**	58 ± 0.9	70.9 ± 0.1
**3**	10.1 ± 0.4	17.6 ± 0.1
**4**	37.3 ± 1.8	47 ± 5.6
**5**	106.8 ± 7.5	97.3 ± 21.1
**6**	18.7 ± 3.6	23.3 ± 3

## Data Availability

Data is contained within the article or Supplementary Materials. The data presented in this study are available in this article and Supplementary Materials.

## Supplementary Materials

The following are available online at https://www.mdpi.com/article/10.3390/cancers13122936/s1. Figure S1: Chemical structure of the compounds validated as Apg2 binders, Figure S2: Apg2 has two intrinsically disordered backbone elongations, as compared to Hsc70, that do not interact with the compounds, Figure S3: Effect of compounds **1–6** on the reactivation of G6PDH aggregates, Figure S4: Cell culture images of controls and compound-treated melanoma cells, Figure S5: The presence of activated caspase-3 is an indicator of apoptosis in PB-treated, melanoma MeWo cells, Figure S6: Time evolution of melanoma cell lines after treatment with PB, Figure S7: Uncropped blots corresponding to data shown in Figure 6B, Figure S8: Interaction of PB with Apg2 and Hsp70.
