# Supplementary material for: Inhibition of the Human Hsc70 System by Small Ligands as a Potential Anticancer Approach
Source: Cancers (Basel). 2021 Jun 11;13(12):2936. doi: 10.3390/cancers13122936 (PMC8230956; doi:10.3390/cancers13122936)
Supplement: Supplementary file 1 [file cancers-13-02936-s001.zip › cancers-1257798-supplementary.pdf]

cancers

MDPI

Article

Supplementary Materials: Inhibition of the Human Hsc70 System by Small Ligands as a Potential Anticancer Approach

Leire Dublang, Jarl Underhaug, Marte I. Flydal, Lorea Velasco-Carneros, Jean-Didier Maréchal, Fernando Moro, Maria Dolores Boyano, Aurora Martinez and Arturo Muga

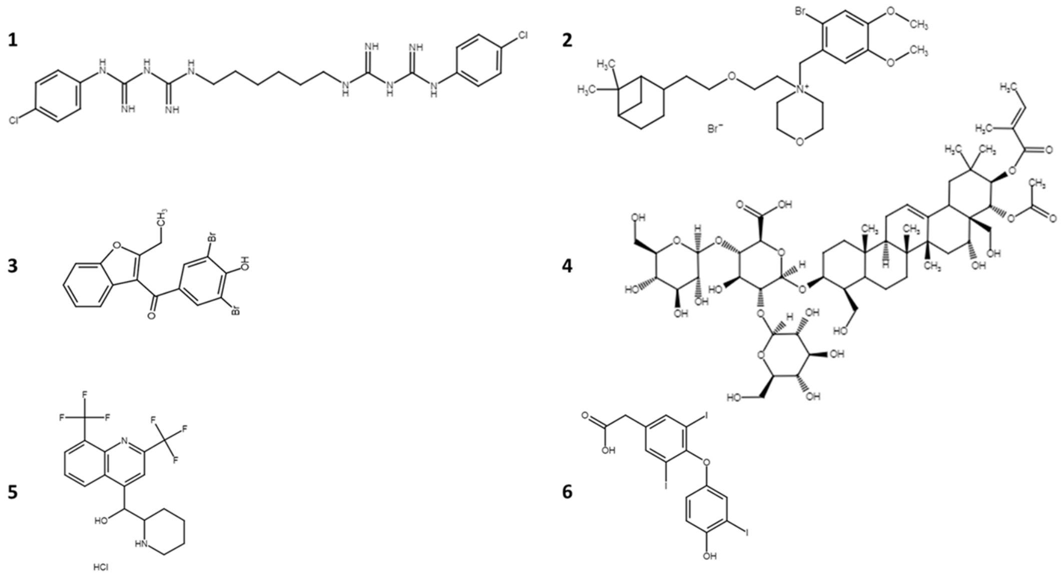

Figure S1. Chemical structure of the compounds validated as Apg2 binders. Names are shown in Table 1.

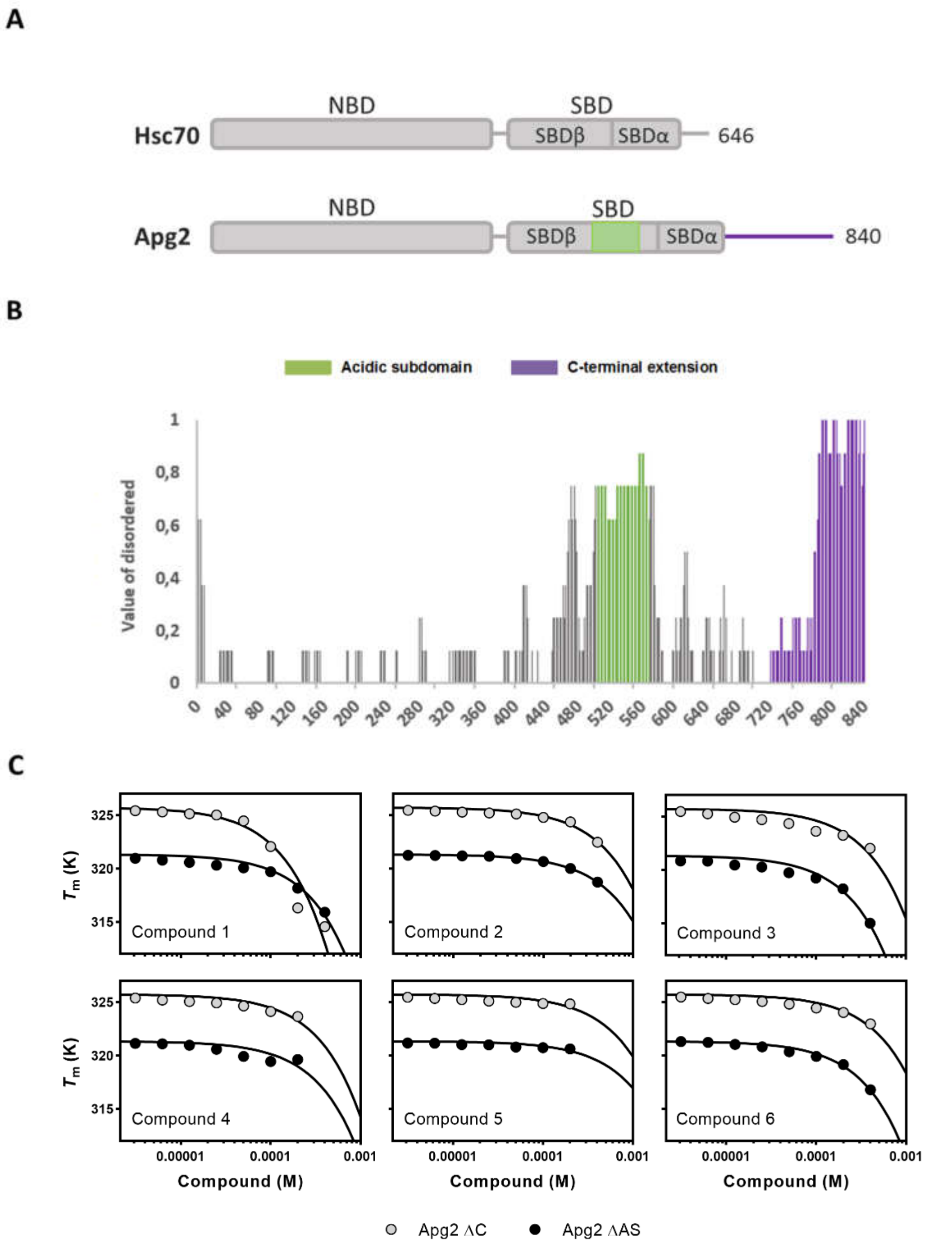

Figure S2. Apg2 has two intrinsically disordered backbone elongations, as compared to Hsc70, that do not interact with the compounds. (**A**) Schematic representation of Hsc70 and Apg2. Both proteins have the same domain distribution: an amino terminal nucleotide binding domain (NBD) and a substrate binding domain split into β and α subdomains. Apg2 has two extensions in the SBD compared to Hsc70: an acidic subdomain inserted in the SBDβ (green) and an extended C-terminal end (purple). (**B**) Intrinsically disordered region (IDR) prediction by MobiDB. Residues are given a value between 0 and 1 as a prediction of disorder. The acidic loop and the C-terminal end are coloured in green and purple, respectively, and correspond to the residues eliminated in each deletion mutant, Apg2 ΔAS and Apg2 ΔC. Both regions are predicted to be disordered. (**C**) Thermal stability of the Apg2 ΔC (grey) and Apg2 ΔAS (black) protein variants in the presence of increasing concentrations of compounds **1–6**. For data fitting see Materials and Methods. The name, molecular weight and chemical structure of these compounds are shown in Table 1 and Figure S1.

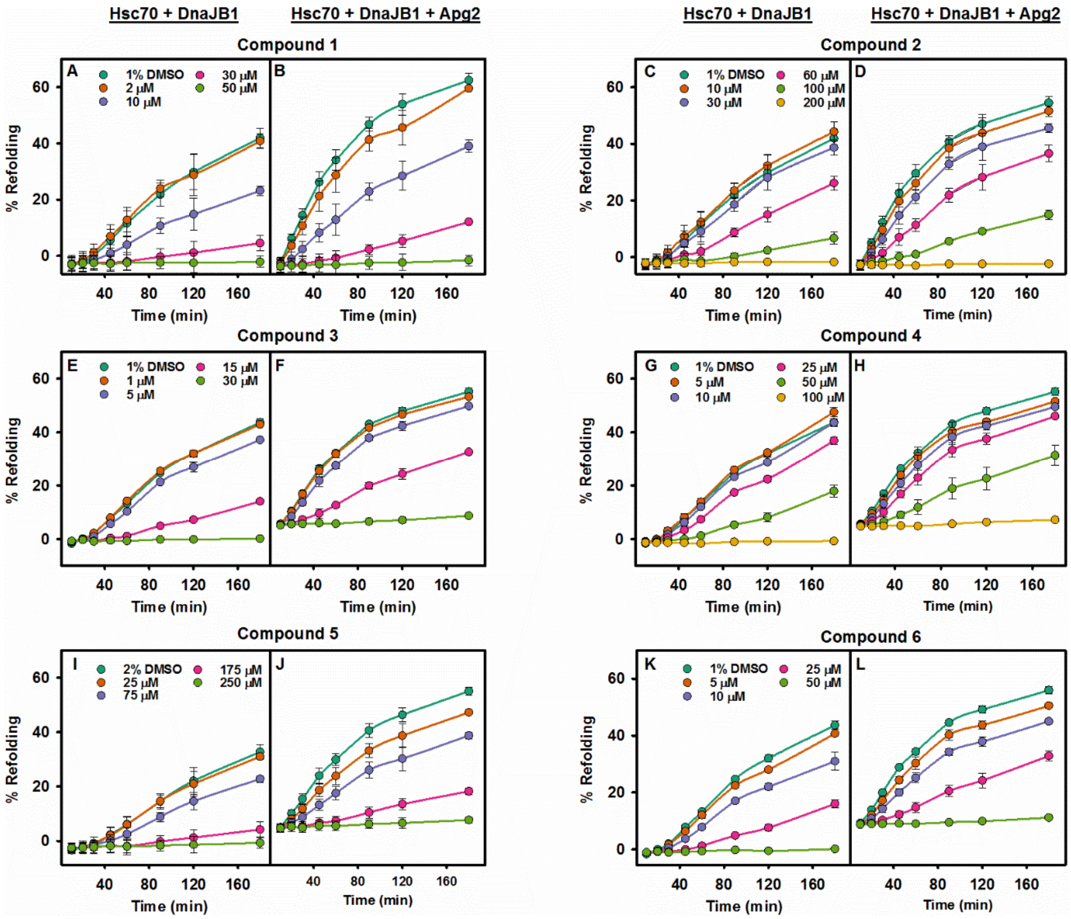

Figure S3. Effect of compounds **1–6** on the reactivation of G6PDH aggregates. Reactivation kinetics of G6PDH aggregates by Hsc70 (2 μM) and DnaJB1 (0.5 μM) in the absence **(A,C,E,G,I,K)** or presence **(B,D,F,H,J,L)** of Apg2 (0.4 μM). Chaperones were incubated with increasing concentrations of compounds **1–6** for 30 min before initiating the reaction by the addition of ATP (2 mM) and aggregated G6PDH (0.4 μM). Values are shown as the means ± SD of 3 independent experiments.

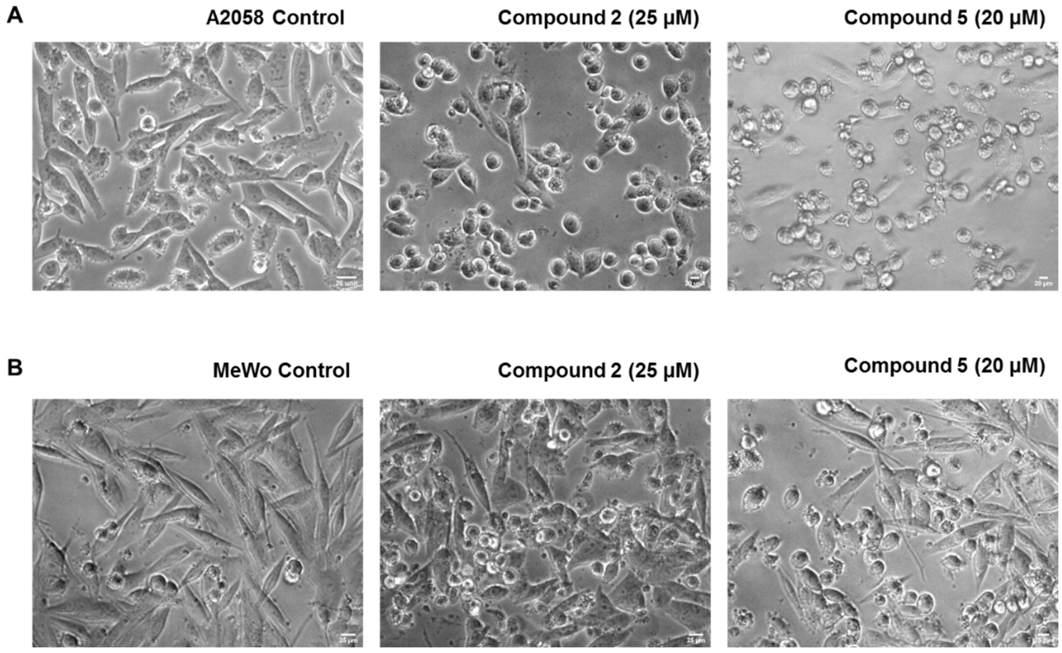

Figure S4. Optical micrographs of cell culture controls and compound-treated melanoma cells. **(A)** A2058 and **(B)** MEWO cell morphology after treatment for 4.5 h with compounds **2** and **5** at the concentrations indicated. Controls show the appearance of the culture in the absence of compounds. Images were taken with a light microscope. Bar 20 μm.

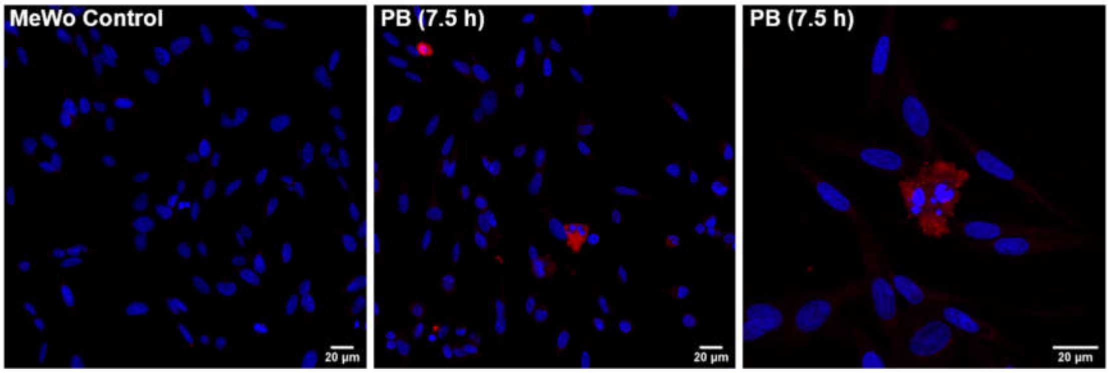

Figure S5. Detection of activated caspase-3 in MeWo cells as an indicator of apoptosis. MeWo cells in the absence (control panel) or presence of 40 μM PB. Samples were stained for activated caspase-3 (red) as an indicator of apoptosis and cell nuclei (blue) were detected with 4′,6-diamidino-2-phenylindole.

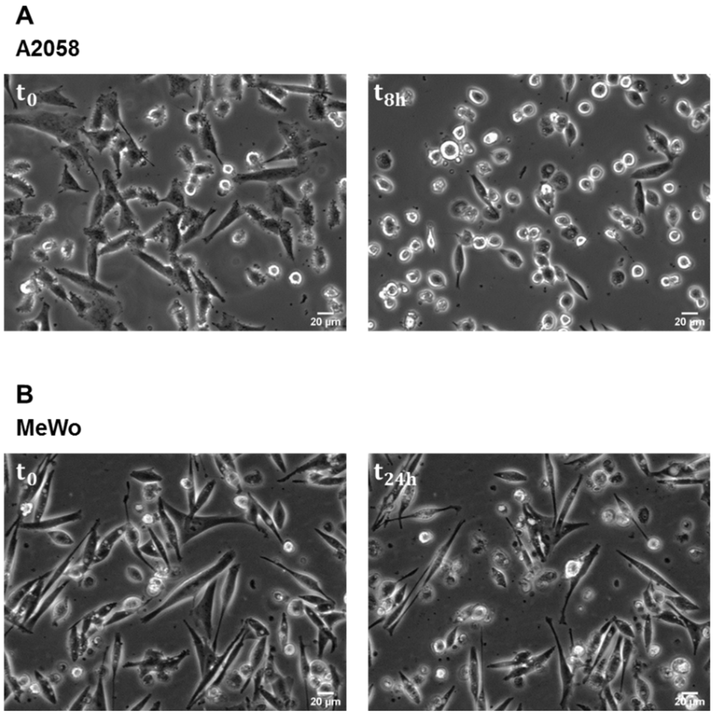

Figure S6. Time evolution of melanoma cell lines after treatment with PB. Snapshots from (**A**) A2058 and (**B**) MeWo melanoma cell lines at the initial time-point (t_0; left panels) and after 8 h (A2058) or 24 h (MeWo) (right panels) incubation with 40 μM PB. Images were taken with a live cell time-lapse microscope. Bar 20 μm.

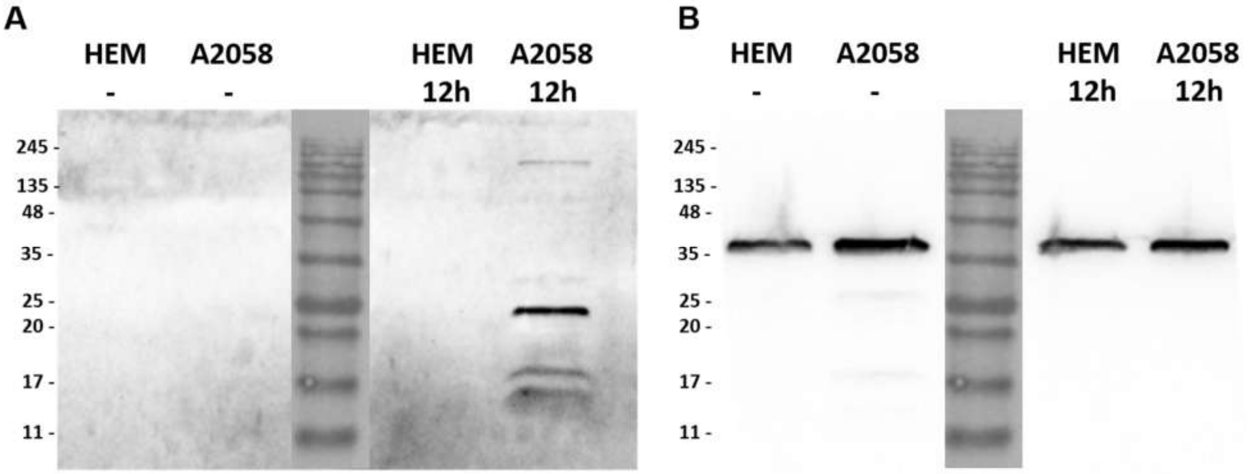

Figure S7. Uncropped blots corresponding to data shown in Figure 6B. (**A**) Immunoblot of caspase-3 corresponding to human melanocytes (HEM) and A2058 cells treated 12 h with 20 μM PB. **(B)** Immunoblot of GAPDH (used as loading control) of the same membrane shown in panel A after mild stripping.

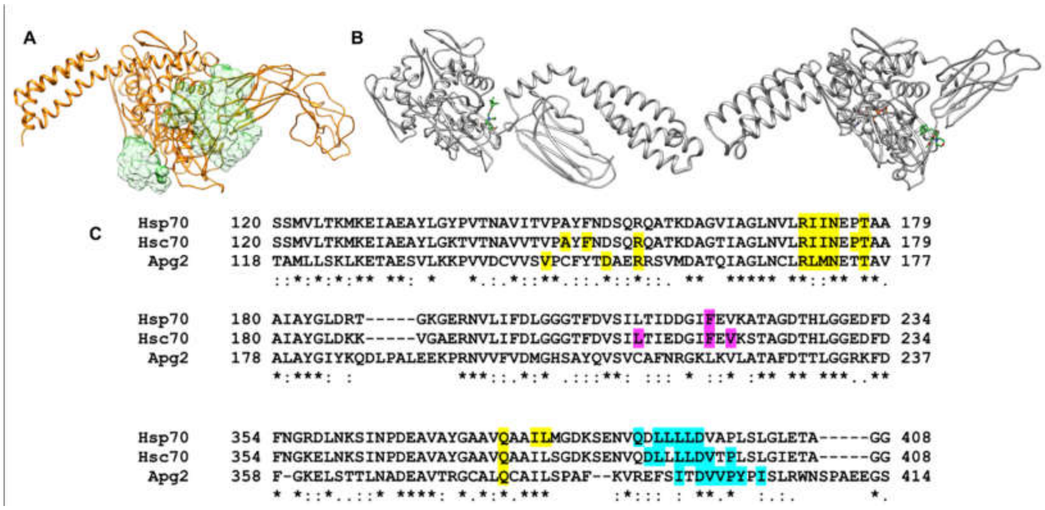

Figure S8. Interaction of PB with Apg2 and Hsp70. **(A)** The two potential binding pockets of PB in Apg2. The green surfaces represent the clusters that PB forms in these regions. The first potential binding region for PB involves mainly the NBD and the linker, whereas the second binding pocket is located in subdomain NBD IA. **(B)** Best docking results of PB (green) on the ADP (left) and ATP (right) states of Hsp70 performed using GOLD. **(C)** Sequence alignment in the NBD and linker regions of human Hsp70 (HSPA1A), Hsc70 (HSPA8) and Apg2 (HSPA4), showing the conserved residues in the NBD IIA (yellow), NBD IA (pink) and linker (blue) regions that interact with PB. Sequence alignment was performed using ClustalW2.
